# Automated F18-FDG PET/CT image quality assessment using deep neural networks on a latest 6-ring digital detector system

**DOI:** 10.1038/s41598-023-37182-1

**Published:** 2023-07-13

**Authors:** Moritz Schwyzer, Stephan Skawran, Antonio G. Gennari, Stephan L. Waelti, Joan Elias Walter, Alessandra Curioni-Fontecedro, Marlena Hofbauer, Alexander Maurer, Martin W. Huellner, Michael Messerli

**Affiliations:** 1 Department of Nuclear Medicine, University Hospital Zurich, Rämistrasse 100, 8091 Zurich, Switzerland; 2 Institute of Diagnostic and Interventional Radiology, University Hospital Zurich, Zurich, Switzerland; 3 University of Zurich, Zurich, Switzerland; 4 Institute of Food, Nutrition and Health, Health Sciences and Technology, ETH Zurich, Zurich, Switzerland; 5 Department of Radiology and Nuclear Medicine, Children’s Hospital of Eastern Switzerland, St. Gallen, Switzerland; 6 Department of Medical Oncology, University Hospital Zurich, Zurich, Switzerland

**Keywords:** Medical research, Molecular medicine, Cancer imaging

## Abstract

To evaluate whether a machine learning classifier can evaluate image quality of maximum intensity projection (MIP) images from F18-FDG-PET scans. A total of 400 MIP images from F18-FDG-PET with simulated decreasing acquisition time (120 s, 90 s, 60 s, 30 s and 15 s per bed-position) using block sequential regularized expectation maximization (BSREM) with a beta-value of 450 and 600 were created. A machine learning classifier was fed with 283 images rated “sufficient image quality” and 117 images rated “insufficient image quality”. The classification performance of the machine learning classifier was assessed by calculating sensitivity, specificity, and area under the receiver operating characteristics curve (AUC) using reader-based classification as the target. Classification performance of the machine learning classifier was AUC 0.978 for BSREM beta 450 and 0.967 for BSREM beta 600. The algorithm showed a sensitivity of 89% and 94% and a specificity of 94% and 94% for the reconstruction BSREM 450 and 600, respectively. Automated assessment of image quality from F18-FDG-PET images using a machine learning classifier provides equivalent performance to manual assessment by experienced radiologists.

## Introduction

2-[^18^F]fluoro-2-deoxy-D-glucose (F18-FDG)-PET is an imaging modality increasingly used for staging various oncological diseases^1^. Image quality in F18-FDG-PET, as well as in other medical imaging modalities, significantly impacts its diagnostic value. Assessment of image quality in PET is commonly achieved using phantoms while applying objective measurements such as number of total photon counts, signal-to-noise ratio (SNR), contrast-to-noise ratio or noise equivalent count rate^2–4^. Some studies have shown a correlation between subjectively perceived image quality and noise equivalent count rate^5^. In clinical practice however, image quality may differ depending on the target lesion studied and ultimately the evaluation remains subjective. Generally in PET, image quality is negatively affected by shorter acquisition time and/or lower administered activity^6^. On the other hand, decreasing injected activity reduces patient and staff radiation exposure and costs, while decreasing scan duration may additionally increase the modality’s availability. An optimization of these parameters specifically requires the capability of fast and reliable image analysis, ideally on-the-fly, while the images are being acquired.

The advent of machine learning in medical imaging has delivered encouraging results, e.g. automated detection of pathology, such as Alzheimer’s disease in F18-FDG-PET data^7^, by massively accelerating otherwise time-consuming segmentation tasks^8^ or in broader terms by linking data collected from medical devices from multiple centers enabling collaborative machine learning without mutual raw data exchange^9,10^. In addition to enabling a timely evaluation of image quality, machine learning has the potential to reduce the notorious subjectivity of the parameter. To our knowledge, a machine learning algorithm has not yet been applied for automated classification of image quality in F18-FDG-PET. Maximum intensity projection (MIP) images serve as a daily driver in the clinical routine enabling physicians to visualize the distribution of F18-FDG and even semi-quantitatively determine the uptake^11^ therefore constituting a primary target for automated image quality classification tasks.

Accordingly, our study aimed to assess the feasibility of using a machine learning algorithm for automated image quality assessment of F18-FDG-PET images in a cohort with simulated decreasing activity.

## Methods

### Study population

This retrospective study included patients undergoing clinically indicated oncologic F18-FDG PET/CT between March and April 2021. Written informed consent for the scientific use of medical data was obtained from all patients. The study was approved by the local ethics committee (Kantonale Ethikkomission Zürich, Zurich, Switzerland). All methods were carried out in accordance with relevant guidelines and regulations.

### Imaging protocol and image reconstruction

Examinations were performed using a latest generation six-ring digital detector PET/CT scanner (Discovery MI Gen 2, GE Healthcare, Waukesha, WI). A clinical 18F-FDG dosage protocol was used, as previously described in detail^12^. Two PET reconstructions were generated using block sequential regularized expectation maximization (Q.Clear, GE Healthcare, Waukesha, WI) with beta-values 450 and 600. To generate PET images with simulated reduced injected 18F-FDG activity, for each patient five sets of PET images were reconstructed using the software supplied with the scanner from identical PET emission data by unlisting “list mode” data, resulting in reduced emission counts equivalent to 120 s, 90 s, 60 s, 30 s and 15 s per bed position. Advantage Workstation Version 4.7 (GE Healthcare, Waukesha, WI) was used to generate maximum intensity projection images in anteroposterior orientation.

### Reader assessment of image quality

Two readers (M.M. and S.S., 9 and 6 years of experience in diagnostic imaging) reviewed all MIP images per patient and assigned each image a subjective image quality score from 1 to 4 (1: non-diagnostic; 2: limited diagnostic value; 3: good diagnostic value; 4: optimal diagnostic value) emulating the assessment done according to the institute’s standard operating procedure and similarly to previous work^13^. One additional reader (A.G.G., 6 years of experience in diagnostic imaging) measured SNRs of PET image datasets by drawing a semi-automated cubicle volume of interest (2 × 2 × 2 cm^3^) in the right liver lobe in the SUV images and dividing the mean standardized uptake value by its standard deviation. The SNR served as a surrogate for objective image quality. Readers were blinded to clinical information.

### Automated image quality assessment using machine learning

For automated image quality assessment, the fast.ai deep learning library^14^ was used in conjunction with a Res-Net-34^15^, a 34-layer residual convolutional network pre-trained on the Image-Net dataset (https://arxiv.org/abs/1512.03385). The core of the classifier was built using the fast.ai vision methods (https://docs.fast.ai/tutorial.vision.html). “CrossEntropyLoss” was chosen as the loss function and “Adam” (https://arxiv.org/abs/1412.6980) was used for gradient-based optimization. For training of the algorithm, 15 learning cycles were set. The library was otherwise empirically used with standard settings as suggested in previous work by Schwyzer et al.^16,17^ who found excellent performance of the algorithm. The MIP datasets from PET images were exported with a total of 400 DICOM files. Each image, according to the readers subjective score, was associated with either a label 1 (= image quality sufficient) when both readers assigned a score greater than 1 or a label 0 (image quality insufficient), when at least one reader assigned a score 1. Imaging sets were split into 10 subsets (8 for training, 1 for validation, 1 for testing) to perform tenfold cross-validation. The results were reported on the test set. Class activation maps were calculated showing which areas of the MIP contributed most to classification. For machine learning computation, a consumer-grade personal computer with an Nvidia GeForce GTX 980 graphics processing unit was used. Performance of the classifier was assessed by calculating the area under the receiver operating characteristic curve (AUC), using reader-based score as target. Sensitivity and specificity were calculated at a cut-off maximizing Youden’s index.

### Statistical analysis

All statistical analyses were performed in the open-source statistics software R (version 4.1.0, R Foundation for Statistical Computing, Vienna, Austria)^18^ including the pROC package. Categorical variables are expressed as frequency distribution and were compared using a Chi-Square-test. Continuous variables are presented as mean ± standard deviation if normally distributed or median (range) otherwise. Assessment of group differences was determined using an unpaired *t*-test after ensuring a normal distribution of the data using the Shapiro–Wilk test. For non-normally distributed data a Wilcoxon-test was used. For all comparisons, a *p*-value of < 0.05 was defined as statistically significant.

## Results

### Study cohort

**Figure 1:**
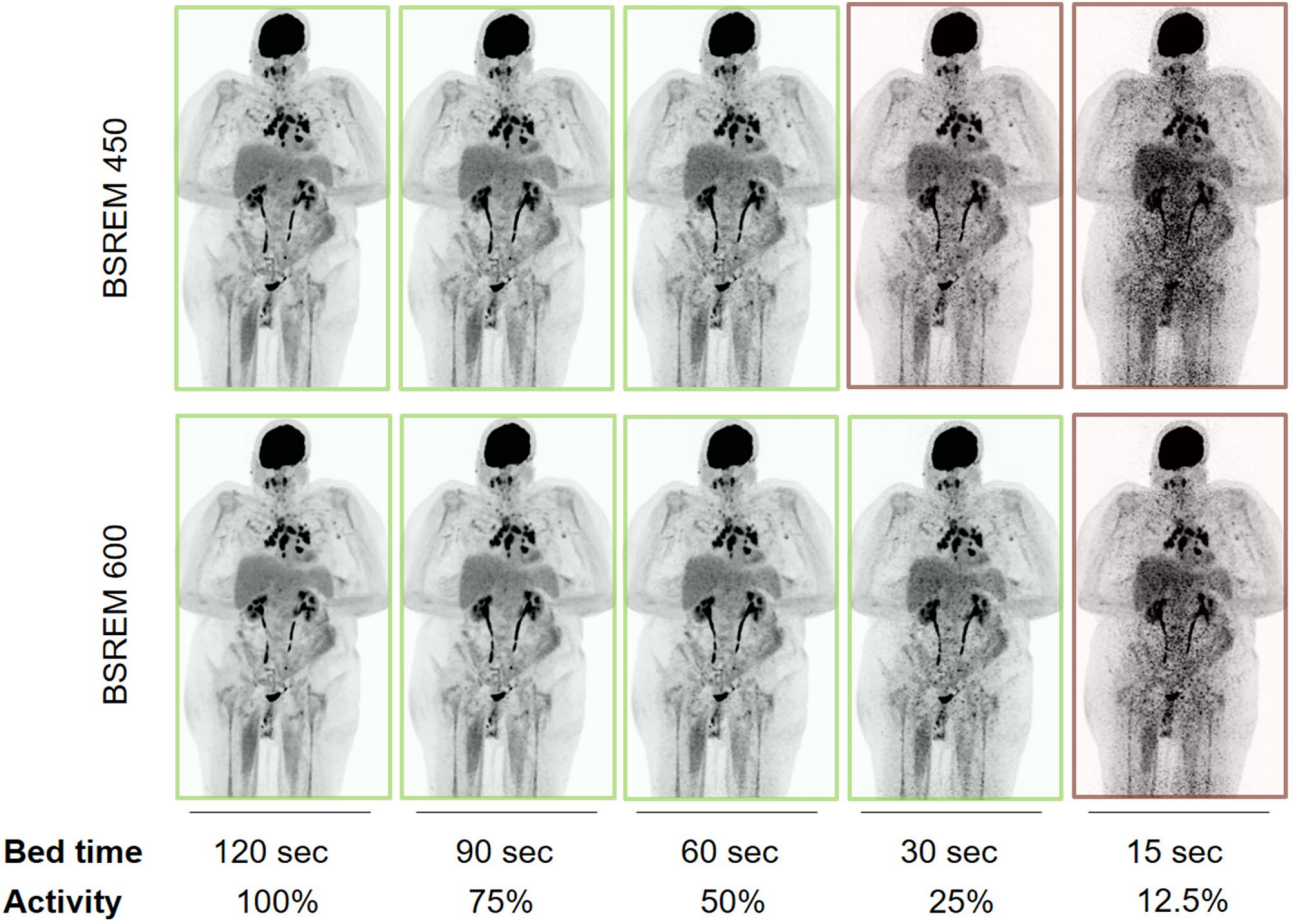
Series of maximum intensity projection PET images with simulated decreasing activity (columns) and the two applied reconstruction setting (rows) using block sequential regularized expectation maximization (BSREM). Images rated sufficient quality by the readers are marked light green, and the images rated as “non-diagnostic” by the readers are marked in light red.

Forty patients were retrospectively included. The mean body mass index was 25.8 ± 5.4 kg/m^2^. The mean injected F18-FDG-activity was 240 ± 60 MBq and images were acquired at 58.7 ± 7.8 min after injection. The most frequent indication for imaging was melanoma (12 / 40, 30%) followed by various squamous cell carcinomas (6 / 40, 15%). Demographic data of the cohort are summarized in Table 1. The algorithm was fed with a total of 400 images (*n* = 283 images with sufficient image quality where a rating of at least “limited diagnostic value” was given by both readers; and *n* = 117 images with insufficient quality where at least one of the two readers assigned the rating “non-diagnostic”). A series of PET images with simulated decreasing activity is given in Fig. 1.

**Table 1:** Demographic data of study subjects (*n* = 40).

Female/male, *n* (%)	15 (37.5%)/25 (62.5%)
Age, years	62 ± 14
Body weight, kg	77.9 ± 19.3
Body height, m	1.73 ± 0.11
BMI, kg/m^2^	25.8 ± 5.4
Blood glucose level at time of injection, mg/dl	101 ± 19
Injected F18-FDG activity, MBq	245 ± 60
PET/CT scan time post injection, min	58.7 ± 7.8
Reason for referral	
Melanoma and other skin cancers	12 (30%)
Various squamous cell carcinomas	6 (15%)
Lung cancer	5 (12.5%)
Breast cancer	5 (12.5%)
Lymphoma	4 (10%)
Laryngeal cancer	2 (5%)
Esophageal cancer	1 (2.5%)
Seminoma	1 (2.5%)
Pancreatic cancer	1 (2.5%)
Sarcoma	1 (2.5%)
Cervical cancer	1 (2.5%)
Colorectal cancer	1 (2.5%)

Values are given as absolute numbers and percentages in parenthesis or mean ± standard deviation.

*BMI* Body mass index, *MBq* Mega-Becquerel, *PET/CT* positron emission tomography/computed tomography.

### Classification performance of the machine learning classifier

**Figure 2:**
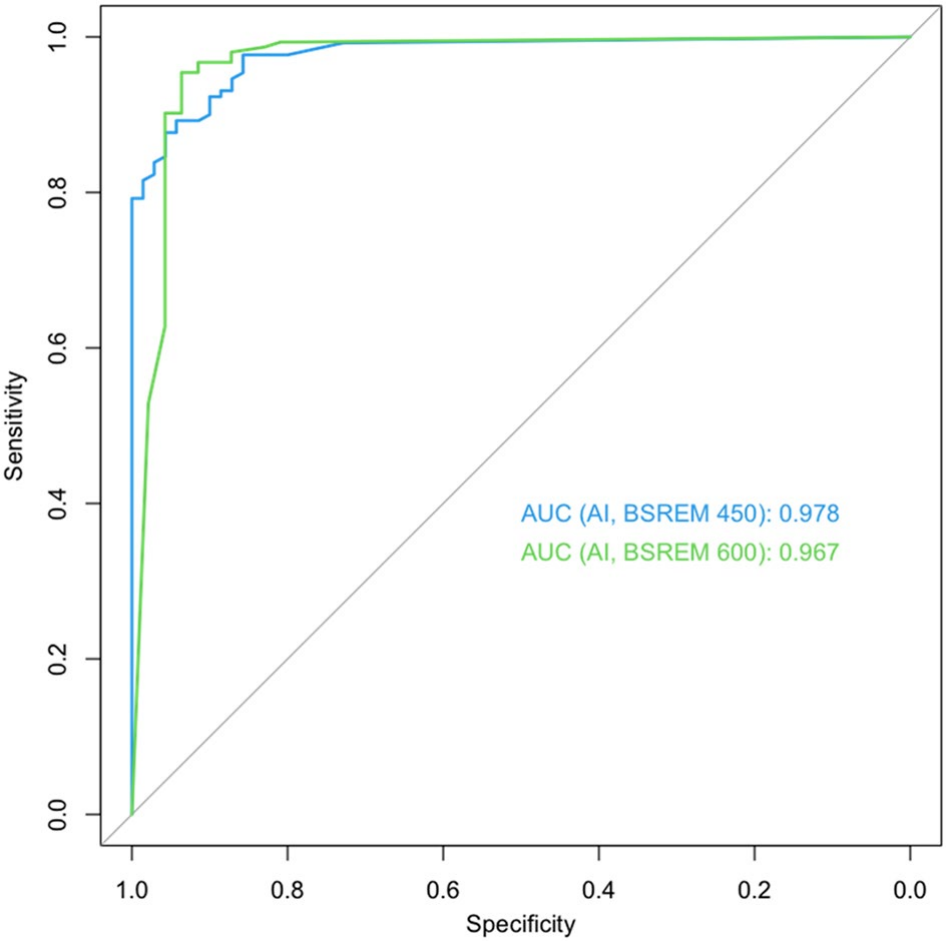
Receiver operating characteristics curves for the performance of image quality classification using the machine learning classifier (AI) grouped by the applied reconstruction setting of block sequential regularized expectation maximization (BSREM) beta 450 and 600, respectively.

The AUC of the machine learning algorithm for the classification of image quality in images reconstructed with BSREM beta 450 was 0.978 (95% Confidence Interval [CI] 0.962–0.994), while for the BSREM beta 600 reconstruction it was 0.967 (CI 0.931–1.000) (Fig. 2). AUCs were not significantly different between the two reconstruction groups (*p* = 0.60). The sensitivities and specificities for the machine learning classifier were 89% and 94% for BSREM beta 450 images and 95% and 94% for BSREM beta 600 images respectively. Among the incorrectly classified images, those with 30 s (i.e., 25%) simulated acquisition dose per bed were most frequently affected (22/80, 15%) while among the images with full (120 s) acquisition time, none were misclassified (Table 2).

**Table 2:** Individual image classification result based analysis of patient and scanning characteristics (*n* = 400).

Characteristics	Correctly classified (*n* = 372)	Incorrectly classified (*n* = 28)	*p* value
Patient age, years	62 ± 13	65 ± 14	0.337
BMI	25.7 ± 5.4	27.0 ± 5.0	0.242
Bed time			
120 s	80 (100%)	0 (0%)	
90 s	78 (97.5%)	2 (2.5%)	
60 s	77 (96.3%)	3 (3.8%)	
30 s	68 (85%)	22 (15%)	
15 s	79 (98.8%)	1 (1.3%)	
Reconstruction algorithm			
Q.Clear 450	182 (91%)	18 (9%)	
Q.Clear 600	190 (95%)	10 (5%)	

Values are means ± standard deviations, or frequencies (percentages).

*BMI* Body mass index, *Q.Clear* block sequential regularized expectation maximization PET reconstruction algorithm.

**Figure 3:**
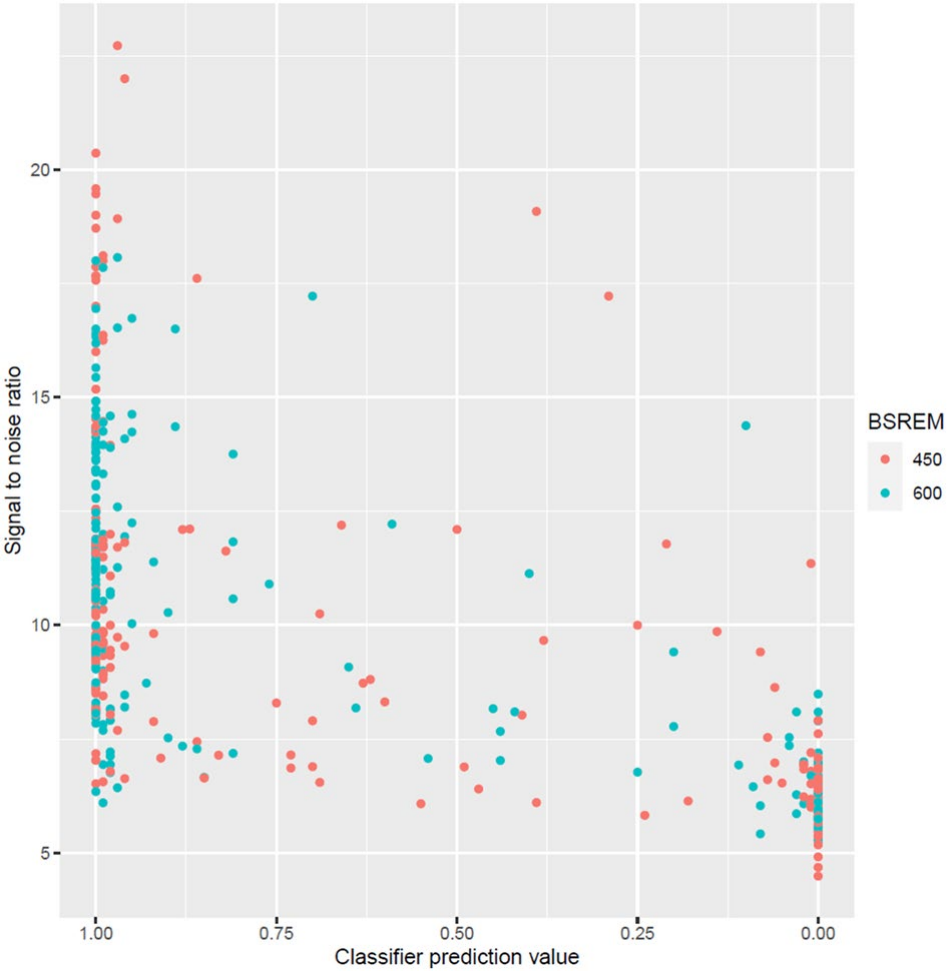
Correlation plot of the machine learning classifiers prediction value and signal to noise ratio measured in liver. Two different reconstruction settings (i.e., block sequential regularized expectation maximization; BSREM) used are marked in red (BSREM beta 450) and green (BSREM beta 600).

SNR measurements in the liver reached an AUC of 0.975 (CI 0.957–0.993) and 0.975 (CI 0.957–0.993) and were similar to the machine learning classifier’s performance (*p* = 0.70). A correlation plot illustrating the relationship between the machine learning classifier’s result and SNR measurements is given in Fig. 3.

**Figure 4:**
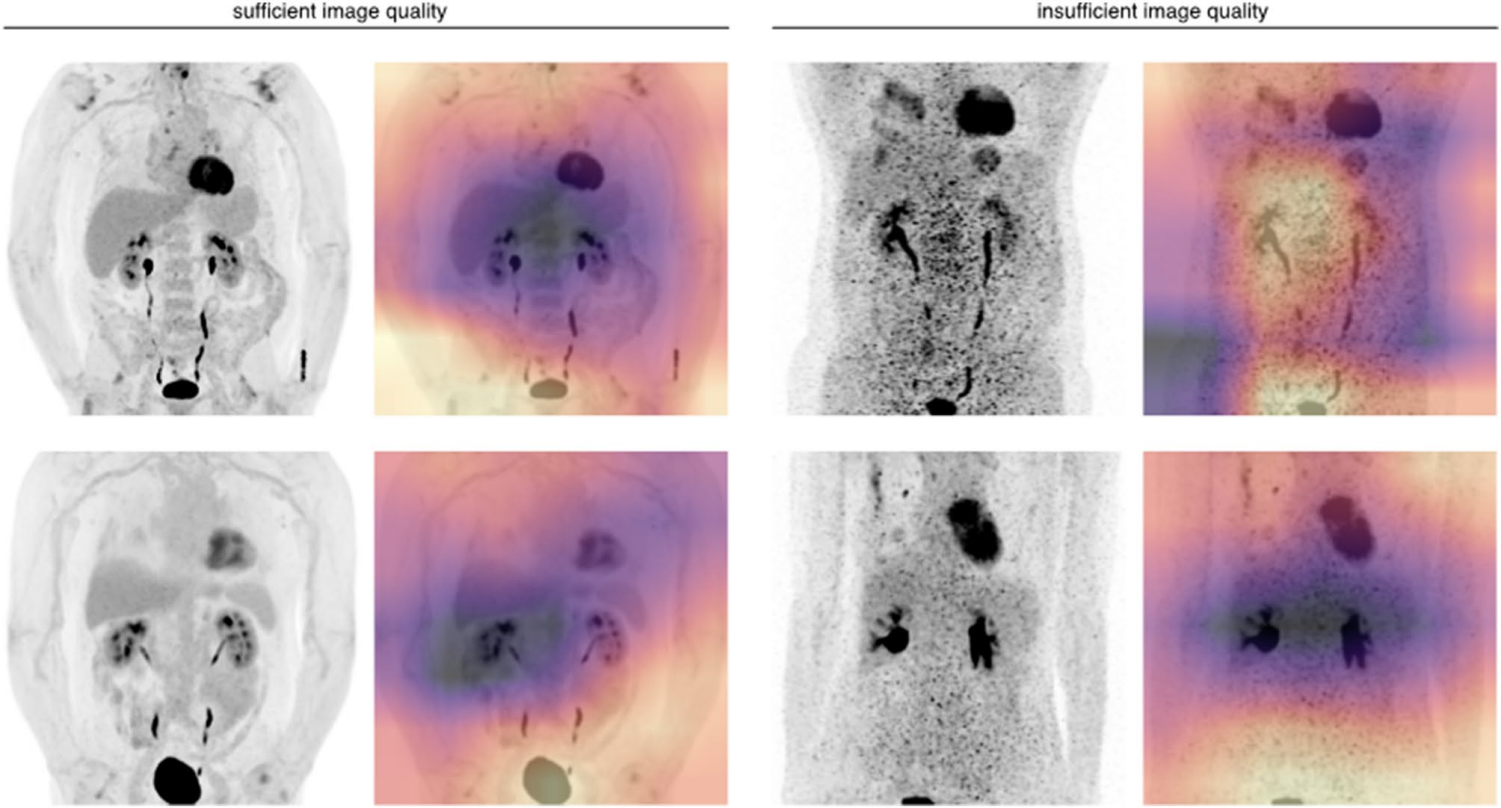
Class activation maps illustrating the machine learning classifier’s targets. Maximum intensity projections are shown with color-coded overlays depicting which parts of the images were most important for classification (yellow areas show high class activation). In images with sufficient image quality (first column) the classifier gets preferentially actived in peripheral areas with low noise. In images with insufficient quality (second column), noisy areas are shown to be most relevant for classification.

To demonstrate targets of the machine learning classifier’s discriminative ability, examples of class activation maps are given in Fig. 4. In cases deemed “non-diagnostic” by the readers, the maps show preferential activation in noisy areas.

## Discussion

In this study, we assessed whether a machine learning classifier can automatically assess the image quality of PET images with simulated decreasing acquisition times resulting in poorer quality. The major findings of our study are as follows: First, machine learning reliably evaluates subjective PET image quality. Second, its classification ability is comparable to manual SNR measurements—a commonly used objective surrogate for image quality. Third, the classifier’s performance did not differ between the different reconstruction settings of BSREM with beta values 450 and 600. Fourth, the classifier most frequently misclassified images in the group of images the experienced readers found to be at the border between non-diagnostic and of limited diagnostic value. This finding further underlines the usefulness of a robust automated classifier in the context of highly subjective visual assessments. Fifth, the classifiers activation maps match noisy areas in non-diagnostic images, ascertaining the validity of the otherwise non-intuitive algorithm.

Previous studies have shown that acquisition times in PET, within certain limits, can be safely reduced while not compromising image quality^19–21^, while others proposed a BMI-based dose regimen^22^ instead of the currently weight-based EANM recommendation^23^. All these suggestions at least partly rely on experienced reader-based evaluation of image quality.

The utility of machine learning in PET has been demonstrated by e.g. detection of F18-FDG-PET-positive nodules and lung cancer^16,17^ and—regarding image quality – very recently by denoising F18-FDG-PET-images post-reconstruction^24^. Yet, it has not been used for estimation of PET image quality itself, a major unmet clinical need in order to perform large-scale image analyses to reduce both dose and scan time while not weakening the diagnostic accuracy of the modality. To our knowledge, our study is the first to exhibit the capability of a candidate machine learning classifier, more specifically deep neural network classifier, aiming to unify a rating task known to be highly subjective. Compared to a radiomics approach used in a previous study including 112 patients the machine learning classifier delivered higher AUCs (AUC 0.978 versus 0.798 in the training and 0.675 in the test dataset^3^).

If more widely applied, it may have an impact on individualized clinical imaging—e.g., building individualized imaging protocols by iterative on-the-fly evaluation of PET images and adjusting acquisition times until a desired image quality is achieved—and research, establishing an agreed-on standard-of-reference for image quality. Moreover, the classifier may be assigned to similar quality assessment tasks in PET imaging whenever qualified subjective judgement is necessary.

Our study has some limitations. Its retrospective scale, the relatively small cohort, and the usage of only one scanner all limit generalizability. Further studies are warranted to ensure wider applicability of our results. Second, image quality assessed in our study is a strictly qualitative and subjective measure. Robustness of quantitative parameters, such as standardized uptake values, needs to be assured by further research. Third, the classifier used in the study was not specifically validated for this task. Further optimization of the classifier might deliver even better performance for evaluation of image quality. Fourth, the standard of reference used to train this algorithm in this study, a reader-based assessment, ultimately remains subjective. To ensure wider applicability and acceptance of an automated assessment, further studies should include a larger number of experienced readers to establish an accepted consensus of image quality.

## Conclusion

Our results suggest that machine learning may be used to automatically evaluate PET image quality. An automated, non-reader dependent machine learning based classifier delivers reliable and almost instantaneous PET image quality assessment. This enhances the clinical workflow significantly by potentially enabling on-the-fly evaluated, individually optimized acquisition protocols with reduced activity and acquisition times.

## Data Availability

The datasets generated during and/or analyzed during the current study are available from the corresponding author on reasonable request.

## Abbreviations

AUC: Area under the receiver operating characteristics curve
CI: 95% Confidence interval
CT: Computed tomography
F18-FDG: 2-[^18^F]fluoro-2-deoxy-D-glucose
MIP: Maximum intensity projection
Q.Clear: Block sequential regularized expectation maximization reconstruction algorithm by GE Healthcare, Waukesha, WI
PET: Positron emission tomography
SNR: Signal-to-noise ratio
